# Utilization of early supported discharge and outpatient rehabilitation services following inpatient stroke rehabilitation

**DOI:** 10.1186/s13690-024-01300-w

**Published:** 2024-05-30

**Authors:** Negar Razavilar, Dat T. Tran, Sean P. Dukelow, Jeff Round

**Affiliations:** 1 Institute of Health Economics, Edmonton, AB Canada; 2 School of Public Health, University of Alberta, Edmonton, AB Canada; 3 Department of Clinical Neurosciences, University of Calgary, Calgary, AB Canada; 4 Faculty of Medicine and Dentistry, University of Alberta, Edmonton, AB Canada

**Keywords:** Early supported discharge, Disability, Stroke rehabilitation, Outpatient stroke rehabilitation

## Abstract

**Background:**

Studies examining factors associated with patient referral to early supported discharge (ESD)/outpatient rehabilitation (OPR) programs and utilization of ESD/OPR services after discharge from inpatient stroke rehabilitation (IPR) are scarce. Accordingly, we examined utilization of ESD/OPR services following discharge from IPR and patient factors associated with service utilization.

**Methods:**

Stroke patients discharged from IPR facilities in Alberta between April 2014 and March 2016 were included and followed for one year for ESD/OPR service utilization. Multivariable linear and negative binomial regressions were used to examine association of patients’ factors with ESD/OPR use.

**Results:**

We included 752 patients (34.4% of 2,187 patients discharged from IPR) who had 40,772 ESD/OPR visits during one year of follow-up in the analysis. Mean and median ESD/OPR visits were 54.2 and 36 visits, respectively. Unadjusted ESD/OPR visits were lower in females and patients aged ≥ 60 years but were similar between urban and rural areas. After adjustment for patient factors, patients in urban areas and discharged home after IPR were associated with 83.5% and 61.9%, respectively, increase in ESD/OPR visits, while having a right-body stroke was associated with 23.5% increase. Older patients used ESD/OPR less than their younger counterparts (1.4% decrease per one year of older age). Available factors explained 12.3% of variation in ESD/OPR use.

**Conclusion:**

ESD/OPR utilization after IPR in Alberta was low and varied across age and geographic locations. Factors associated with use of ESD/OPR were identified but they could not fully explain variation of ESD/OPR use.

**Supplementary Information:**

The online version contains supplementary material available at 10.1186/s13690-024-01300-w.

**Table:** 

Text box 1. Contributions to the literature	
•Currently, little is known about factors associated with patient referral to early supported discharge or outpatient stroke rehabilitation (ESD/OPR) programs and the utilization of ESD/OPR service use after discharge from inpatient rehabilitation (IPR).	
•Our study identified patient demographic and clinical factors (such as residing in urban versus rural areas, being discharged home after IPR, and the presence of certain comorbidities) associated with use of ESD/OPR. These factors did not fully explain variation in ESD/OPR use.	
•Further research and richer data on patients’ clinical and socio-economic characteristics are needed to better understand factors associated with ESD/OPR use among stroke survivors.	

## Introduction

Stroke is one of the leading causes of death and disability in Canada [1, 2]. Approximately 50% of patients who have suffered a stroke live with permanent disabilities [3]. It has been estimated that stroke was associated with 288,427 disability adjusted life years (DALY’s) in Canada in 2016 [2].

Stroke rehabilitation is an important component of post-stroke care to help stroke survivors return to their maximum functional level, develop skills, and live independently. Patients with moderate severity stroke often receive a substantial part of their rehabilitation in inpatient rehabilitation (IPR) facilities. The Canadian Best Practice Recommendations for Stroke Care recommends that stroke patients with ongoing rehabilitation needs should have access to outpatient rehabilitation (OPR) services following discharge [4], and it has been shown that the continuum of care (including emergency response, inpatient acute care, inpatient and outpatient rehabilitation, home-based and community care, and long-term care) for stroke patients results in better patient outcomes [5]. OPR services include hospital based or community based programs [6] and are designed for stroke patients who have continued rehabilitation goals following discharge from acute care or IPR. These services should include the same elements as in IPR services and should be provided for at least 45 min per day per discipline for three to five days per week. The full course of OPR therapy should ideally take at least 8 weeks [7]. Alternative services such as early supported discharge (ESD) have also been developed to facilitate earlier discharge from an acute stroke service or IPR for a select group of patients (that is, patients with mild to moderate disability who are medically stable and have access to appropriate nursing care and other support services such as family/care giver and home services [7]), provide equivalent or improved patient and caregiver outcomes, and reduce healthcare resource use [8–10]. ESD involves a multidisciplinary team of therapists, nurses, and doctors who coordinate through regular meetings to provide services to patients [11]. These services should be provided for at least 5 days per week at the same intensity level as provided through IPR, and if possible, provided by the same medical team that provided IPR to the patient [7]. Studies have established that the most cost-effective method of providing rehabilitation depends on both the types of services available and patient characteristics implying that a single rehabilitation service may not provide equal health and economic benefits for all patients and situations [12, 13]. For example, for some patients, inpatient rehabilitation may be the most cost-effective rehabilitation service; while for other patients, home or community rehabilitation may be the most cost-effective model of care [14].

Studies examining factors associated with patient referral to ESD/OPR programs and utilization of ESD/OPR services after discharge from IPR are scarce. To our knowledge, there has been only one study by Janzen et al. examining factors associated with referral to outpatient services after IPR within the Canadian health care system [13]. The authors suggested that an improved understanding of current practices in OPR is a necessary step towards developing recommendations for streamlining the care continuum and optimizing health care delivery [13]. Accordingly, we conducted a population-based retrospective cohort study of patients with stroke who were discharged from IPR between 2014 and 2016 in Alberta, Canada to examine ESD/OPR utilization, and its association with patient and geographical factors following discharge from IPR. Our study findings could provide better insights into the use of ESD/OPR services and its barriers and facilitators. It could help clinicians, stroke care professionals, and policy makers improve stroke management and care programs.

## Methods

### Data sources and study population

Alberta has a universal coverage and publicly funded health care system that serves a population of more than 4 million people in a large and diverse geographical area. Alberta Health Services (AHS) is the sole healthcare service provider in Alberta and its operation is organized into five geographical health zones (Calgary, Central, Edmonton, North, and South), where Calgary and Edmonton zones are most urbanized and populous (Supplementary Fig. 1) [15]. The ESD/OPR services were successfully piloted in Calgary and Edmonton during 2007–2011 and have been part of the Cardiovascular Health and Stroke Strategic Clinical Network (SCN) in Alberta since 2012. Currently, there are seven ESD/OPR sites in Edmonton, Calgary, Grand Prairie, Camrose, Red Deer, Medicine Hat, and Lethbridge (Agnes Lehman, AHS, personal communication). The ESD/OPR services in Alberta include occupational therapy, physical and physiotherapy, recreation therapy, speech language pathology, psychology, respiratory therapy, social work, and other rehabilitation therapies. These services were provided either face-to-face, at a facility or at the patient’s home, or on the telephone.

We used a previously reported cohort of 2,187 patients who were admitted to 10 IPR facilities in Alberta, Canada between 1 April 2014 and 31 March 2017 (years 2014 to 2016) and survived the IPR episode to identify patients who used ESD/OPR services. Briefly, this IPR patient cohort was those who aged ≥ 18 years, had home as the pre-stroke living setting, were admitted to an IPR facility within 30 days of an acute stroke episode, and discharged alive. This IPR patient cohort was created by linking the National Rehabilitation Reporting System (NRS) [16], Discharge Abstract Database (DAD), and Alberta Health Care Insurance Plan (AHCIP) Registry [17]. Detailed patient selection and characteristics of this IPR patient cohort were described previously [18].

We linked this IPR patient cohort to the ESD/OPR database obtained from AHS, which provides detailed information on a service visit (e.g., facility and date and type of service provided). All patients were followed for one year from the IPR discharge date (index date) for ESD/OPR service utilization. The end date of the follow-up period was 31 March 2018. If a patient had more than one IPR admission during the study period, the last discharge date was used as the index date. Patients who did not have any ESD/OPR visit during one year of follow-up, had rehabilitation services in more than one health zone in Alberta, or did not have active AHCIP coverage (e.g., due to death or emigration) before the end of one year follow-up were excluded.

### ESD/OPR service utilization

We reported ESD/OPR service utilization during one year of follow-up as the number of visits per patient. We examined overall ESD/OPR service use and use by sex, age group, urban/rural residence, and health zones (Calgary, Edmonton, and others). We consulted with rehabilitation physicians and grouped occupational therapy, physical therapy, and physiotherapy together because of their similarities and reported five main groups of services: occupational/physical/physiotherapy, psychology, recreation therapy, social work, and speech-language pathology.

### Statistical analysis

Patient characteristics were summarized using mean (standard deviation [SD]), median (interquartile range [IQR]), count, and percentage, as appropriate. Multivariable linear regression (MLR) with the natural log of the number of visits as the dependent variable was used to examine association of patient and geographical factors with ESD/OPR service use. This method was previously used to study LOS at IPR [18, 19]. Similar to previously reported studies assessing IPR LOS or referral patterns to ESD/OPR services [13, 18, 20], we included patients’ age, sex, body mass index (BMI), FIM score at IPR discharge, comorbidities, residence location, median household income, acute care LOS of the associated acute stroke episode, LOS of the associated IPR admission, year of discharge from IPR, stroke type, stroke position (that is, the side of the body affected by stroke), and health zone [15] in the regression model. We used the likelihood-ratio (LR) test to assess the goodness of fit of the unconstrained model (that is, the model with all patient-level factors) versus the constrained model (that is, the model that only included statistically significant factors). A factor remained in the final constrained model if the LR test results were significant at a 10% level. We did not use the traditional stopping rule of 5% significant level because it has been reported that a strict rule could lead to exclusion of important variables [21–23].

We used previously validated ICD-10 codes to identify patients’ comorbidities [24]. Patients were considered to have the comorbidities in question if the ICD-10 codes corresponding to those comorbidities were recorded in any diagnostic field at admission to IPR, or in any diagnostic field at hospitalizations during the two years prior to IPR admission. The second digit of the patients’ postal code was used to identify their area of residence [25]. All analyses were performed using Stata version 14 (Stata Corporation, College Station, Texas). Two-sided P values < 0.05 were considered statistically significant.

### Sensitivity analysis

In addition to the MLR regression method, we used a multivariable negative binomial (MNB) regression to model the number of ESD/OPR visits as a count variable and ascertain robustness of the regression approach. We included patient-level factors described in the MLR model above and use the LR test to examine inclusion of those factors in the final constrained MNB regression model.

### Ethics approval

This study was conducted as part of a large health evidence review on optimizing stroke rehabilitation practice in Alberta [26], and funded by Alberta Health. Data were provided by Alberta Health subject to the Alberta Health Information Act [27], and approval from a research ethics board was not required.

## Results

**Fig. 1:**
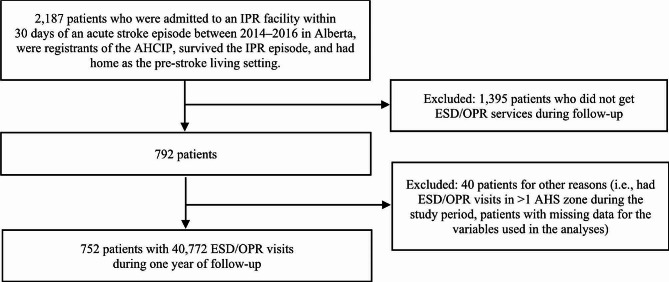
Patient selection flowchart

There were 2,187 patients who had home as their pre-stroke living setting, were admitted to 10 IPR facilities in Alberta between 2014 and 2016, and survived the IPR admission [18]. Of them, 792 (36.2%) used ESD/OPR services during follow-up. After excluding 1,395 (56.2%) patients who did not receive ESD/OPR services during one year post IPR discharge, and 40 patients for other reasons (i.e., patients who had ESD/OPR visits in more than one health zone during follow-up and patients with missing data for the variables used in the analysis), the final study cohort included 752 (34.4%) patients who had 40,772 outpatient rehabilitation visits during one-year post-IPR discharge. A flowchart depicting patient selection is presented in Fig. 1.

Detailed characteristics of the studied population are presented in Supplementary Table 1. Most patients were male (63.6%) and 60 years of age or older (58.8%). Hypertension (76.9%), dyslipidemia (41.1%), and diabetes (29.8%) were the most common comorbidities. Mean acute care LOS of the associated acute stroke episode was 29.4 days, while mean LOS of the associated IPR episode was 58.1 days. FIM score (mean = 109.8) at IPR discharge ranged between 37 and 126. More than 90% of the patients were discharged home following IPR. Most patients (88.9%) lived in urban areas and almost half were in the Calgary zone.

### ESD/OPR service utilization

The average number of ESD/OPR visits per patient was 54.2 (SD = 56.5), and the median number of ESD/OPR visits was 36 (IQR = 12–79). The mean number of visits was lower among female patients compared with male patients (48.7 vs. 57.4 visits, *p* = 0.048) though the median number of visits was similar between the two sex groups (33 vs. 37, *p* = 0.291). The number of visits were higher in patients aged < 60 years (mean = 60.3 visits, < 0.05) compared with older patient groups. There were no differences in ESD/OPR use between urban and rural areas (mean = 54.4 vs. 52.8 visits, *p* = 0.810). Patients in the Calgary zone had the lowest number of visits (mean = 42.2 visits, *p* < 0.05) compared with patients in the other two zone groups (Table 1).

**Table 1:** Number of ESD/OPR visits, by sex, age group, urban residence, and AHS zone

Variable	Mean (SD)	*p*	Median (IQR)	*p*
Sex				
Female	48.7 (47.5)	0.048	33 (13–73)	0.291
Male	57.4 (61.0)		37 (11–83)	
Age group				
18–59 years	60.3 (60.0)	0.003	43 (16–83)	0.006
60–69 years	54.1 (56.6)		37 (10–80)	
70–79 years	46.2 (49.0)		27 (10–73)	
≥ 80 years	41.6 (51.5)		23 (3–51)	
Residence area				
Urban	54.4 (56.1)	0.810	37 (13–77)	0.298
Rural	52.8 (59.7)		25 (6–91)	
AHS zone				
Calgary	42.2 (42.3)	< 0.001	28 (9–66)	< 0.001
Edmonton	56.6 (63.8)		33 (10–79)	
Other	87.9 (63.5)		83 (39–126)	

AHS: Alberta Health Services; ESD: early supported discharge; IQR: interquartile range; OPR: outpatient rehabilitation; SD: standard deviation

The utilization of ESD/OPR services by types and by health zones and urban/rural residency is presented in Tables 2 and 3, respectively. Occupational/physical/physiotherapy was the most used service. Psychology service was only available in Calgary and Edmonton zones, but the utilization was low (0.3 and 4.6 visits per patient in Calgary and Edmonton, respectively). Use of other types of services was lower in Calgary and Edmonton, compared to other zones (all pairwise *p* < 0.05). The mean number of visits to social workers was lower in urban areas (1.2 visits) compared to that in rural areas (2.3 visits; *p* = 0.019), but there were no differences in types of services between urban and rural areas for other four groups of ESD/OPR services (Table 3).

**Table 2:** Utilization of ESD/OPR services, by service type and AHS zone

Service type	Calgary zone		Edmonton zone		Other zones	
	Mean (SD)	Median (IQR)	Mean (SD)	Median (IQR)	Mean (SD)	Median (IQR)
Occupational/physical/physiotherapy	26.0 (26.3)	19 (6–38)	34.3 (43.5)	17 (3–46)	57.1 (46.7)	50 (25–79)
Psychology	0.3 (1.3)	0 (0–0)	4.6 (11.8)	0 (0–3)	-	-
Recreation therapy	2.5 (3.7)	0 (0–4)	9.7 (22.9)	0 (0–9)	15.4 (11.8)	14 (6–24)
Social work	2.0 (3.9)	0 (0–2)	1.4 (4.9)	0 (0–1)	5.4 (5.4)	3 (1–9)
Speech-language pathology	8.1 (15.1)	1 (0–10)	6.8 (15.9)	0 (0–3)	10.1 (9.2)	8 (3–15)

*Note*: There were 363 and 277 patients who received services in Calgary and Edmonton respectively

**Table 3:** Utilization of ESD/OPR services, by service type and urban residence

Service type	Urban		Rural	
	Mean (SD)	Median (IQR)	Mean (SD)	Median (IQR)
Occupational/physical/physiotherapy	33.4 (37.5)	21 (7–47)	34.6 (43.5)	18 (2–66)
Psychology	1.8 (7.6)	0 (0–0)	1.8 (6.9)	0 (0–0)
Recreation therapy	7 (16.3)	1 (0–7)	7.2 (11)	1 (0–12)
Social work	1.2 (3.9)	0 (0–0)	2.3 (5.1)	0 (0–3)
Speech-language pathology	3.8 (11.1)	0 (0–0)	5 (10)	0 (0–8)

ESD: early supported discharge; IQR: interquartile range; OPR: outpatient rehabilitation; SD: standard deviation

The final MLR model predicting the natural logarithm of the number of ESD/OPR visits included age, whether the patient was discharged home after IPR, acute care LOS of the associated acute stroke episode, BMI, urban living location, stroke position, presence of peripheral vascular disease, and health zone. They explained 12.3% of the variation in the frequency of ESD/OPR visits during the study period (Table 4). A one-year increase in age was associated with a reduction in the mean number of visits by 1.4% (*p* = 0.001). Living in an urban (versus rural) area and being discharged home (as opposed to other locations) after IPR were both associated with an increase in the mean number of visits by 83.5% (*p* < 0.001) and 61.9% (*p* = 0.006), respectively. A one-day increase in acute care LOS of the associated acute stroke episode and a one-unit increase in the body mass index were both associated with a slight increase in the mean number of visits by 0.5% (*p* = 0.003) and 0.3% (*p* = 0.034), respectively. Having a right-body stroke (as opposed to left-body) was associated with an increase in the average number of visits by 23.5% (*p* = 0.047). Being in the Edmonton zone and other zones was associated with a higher mean number of visits by 28.5% (*p* = 0.031) and 247.3% (*p* < 0.001), respectively, compared with being in the Calgary zone.

**Table 4:** Predictors of the number of ESD/OPR visits in Alberta, using multiple linear regression (*N* = 752)

Variable	Coefficient [95% CI]	*p*
Age	−0.014 [− 0.022, − 0.006]	0.001
Home discharge	0.482 [0.138, 0.826]	0.006
Urban living location	0.607 [0.290, 0.925]	< 0.001
Acute care LOS of the associated acute stroke episode	0.005 [0.002, 0.009]	0.003
Body mass index	0.003 [0.000, 0.006]	0.034
Stroke position		
Left body (ref)	--	
Right body	0.211 [0.003, 0.420]	0.047
Other^a^	−0.017 [− 0.345, 0.311]	0.918
Peripheral vascular disease	0.399 [− 0.003, 0.800]	0.052
AHS zone		
Calgary (ref)	--	
Edmonton	0.251 [0.023, 0.478]	0.031
Other	1.245 [0.944, 1.547]	< 0.001
Intercept	2.262 [1.525, 3.000]	< 0.001

*Note*: Adjusted R^2^ = 0.123

^a^*Other* includes bilateral involvement, no paresis, and other stroke

AHS: Alberta Health Services; CI: confidence interval; ESD: early supported discharge; LOS: length of stay; *N*: number of patients; OPR: outpatient rehabilitation

### Sensitivity analysis

Table 5 presents detailed results of the MNB regression analysis of ESD/OPR service use. The MNB model is generally consistent with the MLR model, except for the presence of male sex and three comorbidities (hypertension, diabetes, and dyslipidemia) in the MNB. Also, being discharged home following IPR had a significant effect in the MLR but not the MNB model.

**Table 5:** Predictors of the number of ESD/OPR visits in Alberta, using negative binomial regression (*N* = 752)

Variable	IRR [95% CI]	*p*
Male sex	1.198 [1.039, 1.382]	0.013
Age	0.991 [0.984, 0.997]	0.003
Urban living location	1.443 [1.076, 1.936]	0.014
Acute care LOS of the associated stroke episode	1.003 [1.001, 1.005]	0.005
Body mass index	1.00 [0.999, 1.003]	0.088
Stroke position		
Left body (ref)	--	
Right body	1.165 [1.003, 1.353]	0.046
Other^a^	1.086 [0.842, 1.401]	0.525
Peripheral vascular disease	1.276 [0.987, 1.651]	0.063
Hypertension	0.838 [0.695, 1.001]	0.063
Dyslipidemia	1.201 [0.991, 1.456]	0.062
Diabetes	0.857 [0.737, 0.997]	0.045
AHS zone		
Calgary (ref)	--	
Edmonton	1.225 [0.989, 1.518]	0.063
Other	2.460 [1.972, 3.069]	< 0.001
Intercept	33.557 [19.296, 58.358]	< 0.001

*Note*: Pseudo R^2^ = 0.015

^a^*Other* includes bilateral involvement, no paresis, and other stroke.

AHS: Alberta Health Services; CI: confidence interval; IRR: incidence rate ratio; LOS: length of stay; OPR: outpatient rehabilitation.

## Discussion

Using a population-based cohort of patients who were discharged from 10 IPR facilities in Alberta between 2014 and 2016, we found that only 36.2% of the stroke patients used ESD/OPR services during one year after IPR discharge. Overall, use of ESD/OPR services was lower among female and older patients. Patients in the Calgary zone had the lowest number of visits compared with those in Edmonton or other zones. Occupational/physical/physiotherapy was the most used service and there were no differences in types of ESD/OPR services between urban and rural residents, except for visits to social workers. Regression analyses indicated that factors such as age, health zone, acute care LOS of the associated acute stroke episode, BMI, and stroke position, were consistently associated with ESD/OPR service use. The MLR model appeared to be a better fit for our data, with a higher R-squared value than in the MNB model (12% versus 2%, respectively).

The Canadian Stroke Best Practice Recommendations indicate that a patient is a suitable candidate for OPR if the patient’s rehabilitation needs can be met in the community, the patient meets the general inclusion criteria for stroke rehabilitation (as described in the Canadian Stroke Best Practice Recommendations document [4]), the patient is medically stable, ready to participate in rehabilitation, can be accompanied by a caregiver to the therapy sessions if necessary, and can organize transportation to and from the rehabilitation center [4]. While we did not assess factors associated with whether a patient receives ESD/OPR services, patients who did not receive any ESD/OPR services in our study sample may not have been suitable candidates for it based on their medical condition, the patients’ rehabilitation needs could not be met in the community, and/or the patients may not have had the required assistance (if needed) for participation in ESD/OPR.

Studies examining the factors that determine transitions from IPR to community/outpatient stroke rehabilitation among stroke patients, including which patients get referred to ESD/OPR and which referred patients attend the therapy program, are scarce. Sandel et al. 2009, studied the demographic, socioeconomic, and geographical disparities in access to a variety of post-acute stroke rehabilitation services (including inpatient rehabilitation hospital [IRH], skilled nursing facility [SNF], home health care [HH] and outpatient, or no rehabilitation services) during the year after stroke in the United States, but did not specifically examine the factors associated with the receipt/number of visits of outpatient stroke rehabilitation following receipt of IPR services. They found that the percentage of individuals in the SNF and HH categories as the highest utilized post-acute care service categories decreased between 1996 and 2003, while the percentage of individuals in the outpatient services category as the sole post-cate care treatment increased over time [28]. Freburger et al. [29] also found demographic differences in post-acute rehabilitation care (that is, receiving HH versus no HH among those discharged to home, and receiving IRH versus SNF among those discharged to institutions) among patients in selected states in the United States, even after controlling for factors such as illness severity, comorbidities, and supply. More specifically, they found that Blacks, women, older individuals, and lower income individuals were more likely to get discharged to an institution versus home, while Hispanics and the uninsured were less likely to receive institutional care. Conditional on being discharged home, racial minorities, women, older individuals, and lower income individuals were more likely to receive HH than no HH, while the uninsured were less likely to receive it. Chan et al. [30] used the same cohort used by Sandel et al. to examine disparities associated with the number of outpatient rehabilitation visits during the year following discharge from acute care. Similar to our findings, Chan et al. found age to be negatively associated with the number of outpatient visits and the acute care LOS to be positively associated with the number of visits. However, the study did not specifically examine the number of outpatient rehabilitation visits among those who had received IPR services. They also did not include patient comorbidities in their analysis, which were found to be highly correlated with the number of ESD/OPR visits in the present study. Further, we used two different models to examine the factors associated with the number of ESD/OPR visits and found consistent results. It should be noted that studies by Sandel et al., Freburger et al., and Chan et al. used United States data, so there may be variations between the results of these studies and the present study due to the differences between the United States and Canada healthcare systems as reported previously [31].

Janzen et al. performed a retrospective chart review of a cohort of 1,497 stroke patients who were from an IPR facility between 1 January 2009 and 1 March 2016 within the Southwest Local Health Integration Network geographical boundaries in Ontario [13]. Among these patients, 891 were referred to an OPR program, and 721 of these attended the program. Those who were referred were significantly younger, had higher FIM scores at discharge, and had shorter IPR LOS compared with those who were not referred. Also, most of the referred patients were discharged home following IPR. Patients who attended the program (that is, patients who received the OPR therapy) were, again, significantly younger and had higher discharge FIM scores but did not have a significantly different stroke severity compared with those who did not attend the program. In addition, among patients who received OPR therapy, the average number of visits was 32.2 (standard deviation: 26.2). Our study differs from Janzen et al. in that we examined the factors that were associated with the utilization of ESD/OPR services (by those who received any service). We also looked at a wider range of patient factors than Janzen et al., including more detailed patient characteristics (specifically for predicting the frequency of visits). Unlike Janzen et al. who found that getting referred to OPR services and/or receiving any OPR services was significantly associated with the discharge FIM score and IPR LOS, we did not find any of those factors to be significantly associated with the utilization of ESD/OPR services received. Instead, we found the LOS of the associated acute stroke episode to have a positive and significant effect on the number of ESD/OPR visits. However, low R-squared value of 12% suggests that there could be other factors that we did not observe in the data and hence could not control for. Further research and richer data on patients’ clinical and socio-economic characteristics are needed to fill this gap.

Optimal allocation of healthcare resources between acute care and rehabilitation, and among segments of rehabilitation including inpatient rehabilitation, OPR, and ESD is another key challenge for healthcare planners in responding to the increasing demand for provision of care to stroke survivors [32]. Yan et al. used a stroke rehabilitation optimal model, combining discrete event simulation with a genetic algorithm, that changes care capacity across segments of rehabilitation to identify an optimal solution for minimizing wait times in each segment in Alberta. Their model predicted that if ESD and OPR could be provided to additional 138 and 262 stroke survivors, respectively (compared with the status quo), it would result in cost savings of $25.45 million annually [32].

Another challenge in the delivery of post-stroke rehabilitation services is in their delivery to patients residing in rural settings as they have been shown to have decreased access to healthcare, including rehabilitation services, compared with those residing in urban areas [33, 34]. This may partly explain our finding regarding a higher utilization of ESD/OPR services among urban residents than rural residents. Allen et al. suggested providing home-based specialized rehabilitation services for rural residents as a potential solution to this problem and found that providing this service will result in functional gains for rural resident comparable to those living in urban settings [35]. However, successful home-based rehabilitation partly depends on effective communication and collaboration between the caregiver, patient, and therapist [36, 37]. Fisher et al. also suggested that (a) developing strategic networks can help understand the needs of these patients at an organizational level and (b) the existing gap in skill mix and staff establishment among teams providing rehabilitation services is one reason for the unmet needs of patients with more severe disabilities [38].

Although this study contributes to knowledge of the clinical and socio-demographic factors associated with utilization of ESD/OPR services following discharge from IPR among stroke patients, it has limitations. The administrative data sets did not include several clinical data elements of the acute stroke episode [18]. These clinical data, such as the severity of specific impairments (that is, ataxia or aphasia) which may not be fully captured in the FIM score, can be associated with increased LOS at IPR [39]. We expect these factors to contribute to the number of ESD/OPR visits following discharge from IPR as well. Thus, more detailed information about the patients’ clinical characteristics could facilitate better understanding about the association between those factors and the utilization of ESD/OPR services received.

## Conclusion

Our population-base cohort study of patients with inpatient stroke rehabilitation found that utilization of early support discharge and outpatient rehabilitation in Alberta was low and it varied according to patient sex, age, area of residence and service type. Factors associated with utilization of outpatient rehabilitation were generally consistent with those reported in the literature, suggesting that our study findings could be considered in other jurisdictions with similar health care systems. Future research with additional clinical data is warranted to further improve understanding of outpatient stroke rehabilitation and support better care for patients with stroke.

### Electronic supplementary material

Below is the link to the electronic supplementary material.

Supplementary Material 1

## Data Availability

The data that support the findings of this study are available from Alberta Health. However, restrictions apply to the availability of these data, which were used under license for the current study, and so are not publicly available. Data are, however, available from the authors upon reasonable request and with permission from Alberta Health.
