# Supplementary material for: Utilization of early supported discharge and outpatient rehabilitation services following inpatient stroke rehabilitation
Source: Arch Public Health. 2024 May 30;82:80. doi: 10.1186/s13690-024-01300-w (PMC11137928; doi:10.1186/s13690-024-01300-w)
Supplement: Supplementary file 1 — Supplementary Material 1 [file 13690_2024_1300_MOESM1_ESM.docx]

**Supplementary Material**

Supplementary Figure 1: Alberta Health Services Zone Map

Source: https://www.albertahealthservices.ca/ahs-map-ahs-zones.pdf

Supplementary Table 1. Characteristics of the studied population

| **Variable** | **All patients** |
| --- | --- |
| Patients, *N* | 752 |
| ESD/OPR visits, mean (SD) | 54.2 (56.5) |
| ESD/OPR visits, median (IQR) | 36 (12-79) |
| Female sex, n (%) | 274 (36.4) |
| Age group, *n* (%) |  |
| 18–59 years | 310 (41.2) |
| 60–69 years | 240 (31.9) |
| 70–79 years | 154 (20.5) |
| ≥80 years | 48 (6.4) |
| Urban living location, *n* (%) | 669 (88.9) |
| Comorbidities, *n* (%) |  |
| Hypertension | 579 (76.9) |
| Dyslipidemia | 309 (41.1) |
| Diabetes | 224 (29.8) |
| Atrial fibrillation | 127 (16.9) |
| Chronic pulmonary disease | 83 (11.0) |
| Myocardial infarction | 73 (9.7) |
| Peripheral vascular disease | 46 (6.1) |
| Cancer | 44 (5.9) |
| Heart failure | 36 (4.8) |
| Renal disease | 31 (4.1) |
| Rheumatoid disease | 13 (1.7) |
| Liver disease | 7 (0.9) |
| Peptic ulcer | 6 (0.8) |
| Dementia | 5 (0.7) |
| Metastatic cancer | 4 (0.5) |
| IPR LOS in days, mean (SD) | 58.1 (54.1) |
| Discharge FIM score, mean (SD) | 109.8 (13.0) |
| Stroke position, *n* (%) |  |
| Left body | 292 (38.8) |
| Right body | 367 (48.8) |
| Other^a^ | 93 (12.4) |
| Body mass index, mean (SD) | 28.5 (32.7) |
| Home discharge | 685 (91.1) |
| AHS zone, *n* (%) |  |
| Calgary | 363 (48.3) |
| Edmonton | 279 (37.1) |
| Other | 110 (14.6) |
| Acute care LOS of the associated acute stroke episode, mean (SD) | 29.4 (30.5) |
| Household income in $, n (%) |  |
| 0–40,000 | 26 (3.5) |
| 40,000–60,000 | 85 (11.3) |
| 60,000–80,000 | 136 (18.1) |
| 80,000–100,000 | 163 (21.7) |
| >100,000 | 342 (45.5) |
| Year of discharge from IPR, *n* (%) |  |
| 2014 | 218 (29.0) |
| 2015 | 261 (34.7) |
| 2016 | 273 (36.3) |
| Stroke type, *n* (%) |  |
| Ischemic | 578 (76.9) |
| Hemorrhage | 148 (19.7) |
| Other | 26 (3.5) |

^a^ *Other* includes bilateral involvement, no paresis, and other stroke.

FIM: Functional Independence Measure; IQR: interquartile range; IPR: inpatient rehabilitation; LOS: length of stay; SD: standard deviation.
